# A Random Walk-Based Method to Identify Candidate Genes Associated With Lymphoma

**DOI:** 10.3389/fgene.2021.792754

**Published:** 2021-11-25

**Authors:** Minjie Sheng, Haiying Cai, Qin Yang, Jing Li, Jian Zhang, Lihua Liu

**Affiliations:** ^1^ Department of Ophthalmology, Yangpu Hospital, School of Medicine, Tongji University, Shanghai, China; ^2^ Department of Ophthalmology, Shanghai General Hospital, Shanghai Jiao Tong University School of Medicine, Shanghai, China; ^3^ Shanghai Key Laboratory of Ocular Fundus Diseases, Shanghai, China; ^4^ Shanghai Engineering Center for Visual Science and Photomedicine, Shanghai, China; ^5^ National Clinical Research Center for Eye Diseases, Shanghai, China; ^6^ Shanghai Engineering Center for Precise Diagnosis and Treatment of Eye Diseases, Shanghai, China

**Keywords:** lymphoma, random walk with restart algorithm, protein-protein interaction network, enrichment theory, permutation test

## Abstract

Lymphoma is a serious type of cancer, especially for adolescents and elder adults, although this malignancy is quite rare compared with other types of cancer. The cause of this malignancy remains ambiguous. Genetic factor is deemed to be highly associated with the initiation and progression of lymphoma, and several genes have been related to this disease. Determining the pathogeny of lymphoma by identifying the related genes is important. In this study, we presented a random walk-based method to infer the novel lymphoma-associated genes. From the reported 1,458 lymphoma-associated genes and protein–protein interaction network, raw candidate genes were mined by using the random walk with restart algorithm. The determined raw genes were further filtered by using three screening tests (i.e., permutation, linkage, and enrichment tests). These tests could control false-positive genes and screen out essential candidate genes with strong linkages to validate the lymphoma-associated genes. A total of 108 inferred genes were obtained. Analytical results indicated that some inferred genes, such as *RAC3*, *TEC*, *IRAK2/3/4*, *PRKCE*, *SMAD3*, *BLK*, *TXK*, *PRKCQ*, were associated with the initiation and progression of lymphoma.

## 1 Introduction

Lymphocytes are a group of effective immune-associated cells and include two famous cell subtypes, namely, T and B lymphocytes (Mesquita Júnior et al., 2010). Lymphocytes play an irreplaceable role in humoral (B lymphocytes) and cellular (T lymphocytes) immune responses (Mesquita Júnior et al., 2010) to fight against infectious virus or bacteria and endogenous malignant cancer cells. However, even as immune cells, lymphocytes can also be malignant when transformed by exogenous stimulations, such as benzene (Guo et al., 2021) or the human immunodeficiency virus (Wang et al., 2021), and endogenous factors, such as family history (Chang et al., 2005) and aging (Parsonnet and Isaacson, 2004). Cancers that begin in the immune-associated lymphocytes are generally summarized as lymphoma (Armitage et al., 2017).

Lymphoma can be divided into two groups, namely, Hodgkin lymphoma (Mathas et al., 2016) and non-Hodgkin lymphoma (Shankland et al., 2012) according to the existence of reed-sternberg cells. Lymphoma with and without detectable reed-sternberg cells are generally regarded as Hodgkin and non-Hodgkin lymphoma (Shankland et al., 2012; Mathas et al., 2016). Both kinds of lymphoma are quite rare compared with other cancer subtypes, such as lung and liver cancers (Siegel et al., 2021). Approximately 9,000 new cases and 1,000 deaths have been reported in 2020 by the American Cancer Society (Siegel et al., 2021). Contrary to other cancer subtypes, the risk of lymphoma is quite high for adolescents and elder adults (older than 55 years old) but relatively low for adults in their 30 and 40 s (Wilson et al., 2012). This characteristic reflects a typical age-associated disease susceptibility distribution pattern for lymphoma.

However, the cause of non-Hodgkin lymphoma remains unknown. Several reports have associated some viruses, such as *T cell leukemia lymphoma virus* (Zhang et al., 2017b), Epstein-Barr virus (Vockerodt et al., 2015), and hepatitis B virus (Ren et al., 2018), and bacteria, such as *Helicobacter pylori* (specific for gastric MALT lymphoma) (Salar, 2019), with the pathogenesis of non-Hodgkin lymphoma. For the Hodgkin lymphoma, the risk is increased in people with Human Immunodeficiency Virus and Epstein-Barr virus infections (Grewal et al., 2018). For both types of lymphoma, family history has long been considered as an important risk factor, and genetic background has also been highly associated with the initiation and progression of this cancer (Skibola et al., 2007). According to a review for the genetic susceptibility to lymphoma, seven groups of genes with the following functions are involved in the pathogenesis of lymphoma as follows: DNA repair [e.g., *NHEJ* (Lieber et al., 2010) and *DSBR* (Shen et al., 2006)]; carbon metabolism [e.g., *MTHFR* (He et al., 2014) and *MTR* (Ruiz-Cosano et al., 2013)]; immune regulation [e.g., *TNF*, *IL4*, and *IL4R* (Mottok and Steidl, 2015)]; oxidative stress [e.g., *NOS2A* (Fabisiewicz et al., 2013) and *MPO (* *Sugiyama et al., 2017* *)*]; energy regulation [e.g., *LEP* and *GHRL* (Argyrou et al., 2019)]; hormone production [e.g., *CYP17A1 (* *Skibola et al., 2005* *)*]; xenobiotic [e.g., *GSTT1* (Yang et al., 2014)]; and cell cycle regulation [e.g., C*CND1* (Mohanty et al., 2019)]. The association of these genes with the pathogenesis of lymphoma has been established. Thus, the initiation and progression of lymphoma are precisely regulated by genetic background. Finding the genetic factors for lymphoma is therefore one of the most effective and straight-forward approaches to reveal the pathogenesis of such complex diseases.

Traditionally, the identification of lymphoma associated genes depends on several classical analytic approaches and methods. For familial lymphoma cases, family pedigree analyses based on Sanger sequencing (Liu et al., 2014), microarray analyses (Hedvat et al., 2002), next generation target sequencing and whole genome wide sequencing (Hung et al., 2018) on large familial samples are major traditional methods to identify potential pathogenic lymphoma associated genes or variants. As for sporadic lymphoma cases, to validate the molecular abnormalities associated with lymphoma, Southern blot analyses (Sangueza et al., 1992), *in situ* hybridization (Quintanilla-Martinez et al., 2009) and quantitative real-time PCR (Takatori et al., 2021) are also applied to explore and confirm specific distribution of genetic abnormal arrangement associated with lymphoma. There are three advantages for traditional analyses: 1) Firstly, the accuracy of traditional experimental analyses is generally higher than statistical bioinformatics analyses; 2) Secondly, independent repeat experimental analyses are easier to perform at experimental level to validate the identified potential biomarkers; 3) Thirdly, results from experimental analyses were easier to be used for further functional exploration. However, the disadvantages of experiment-based analyses are also obvious, including 1) Clinical samples are difficult to obtain, and results from experimental animals are not always consistent with human beings; 2) Low reproducibility caused by more potential unrelated variables; 3) High cost and time consuming.

Due to the high cost and time consuming of traditional experiment-based methods, we introduced a random walk-based computational method to recognize the novel candidate lymphoma-associated genes in this study. The reported lymphoma-associated genes, as summarized from the DisGeNET database (Piñero et al., 2015), and the protein–protein interaction (PPI) network collected in STRING (Szklarczyk et al., 2015), were fed into the random walk with restart (RWR) algorithm (Kohler et al., 2008; Macropol et al., 2009) to determine the raw candidate genes. Then, three screening tests (i.e., permutation, linkage, and enrichment tests) were performed to control false-positive genes and select the essential candidate genes that had strong linkages to validate the lymphoma-associated genes. The analytical results indicated that several of these genes had associations with the initiation and progression of lymphoma.

## 2 Materials and Methods

### 2.1 Lymphoma-Associated Genes

In this study, we summarized all lymphoma-associated genes from the DisGeNET database (https://www.disgenet.org/, version 7.0, accessed in March 2021) (Piñero et al., 2015), one of the largest publicly available databases of human genes and gene associated with human diseases. A total of 1,548 genes have been associated with the pathogenesis of lymphoma in the past 5 years (Supplementary Table S1). Then, the related proteins of these genes were picked up and further mapped onto their Ensembl IDs. The IDs not in the PPI network as described in Section 2.2 were excluded, resulting in 1,375 Ensembl IDs. Based on these proteins, as represented by Ensembl IDs, we set up a computational method to discover other proteins, which were highly related to these proteins. The genes encoding the identified proteins were regarded to be highly associated with the pathogenesis of lymphoma.

### 2.2 PPI Network

This study proposed a random walk-based method to investigate the lymphoma-associated genes. A network should be employed to execute the random walk algorithm. In recent years, the PPI network is widely used to study various problems related to proteins or genes (Ng et al., 2010; Hu et al., 2011a; Hu et al., 2011b; Zhang et al., 2016; Cai et al., 2017; Zhang et al., 2019; Zhang and Chen, 2020; Zhao et al., 2020; Gao et al., 2021). Thus, we used the structure of one PPI network and mined new candidate genes related to lymphoma based on the validated ones.

We employed the PPI network collected in STRING (version 10, https://www.string-db.org/) (Szklarczyk et al., 2015). The file “9,606. protein.links.v10. txt.gz” was retrieved, which consisted of 4,274,001 PPIs covering 19,247 human proteins. A PPI included two proteins, encoded by Ensembl IDs. Furthermore, one confidence score with range between 1 and 999 was assigned to each PPI. Such score can comprehensively measure the associations of proteins, because it integrates several scores, including “neighborhood”, “fusion”, “cooccurence”, “coexpression”, “experimental”, “database”, and “textmining” scores, which assess the associations of proteins from various aspects of proteins, such as close neighborhood in (prokaryotic) genomes, gene fusion, occurrence across species, gene coexpression, scientific literature description, etc. The higher the score was, the stronger the PPI would be. Accordingly, a PPI network was constructed by taking 19,247 human proteins as nodes, and two nodes were connected by an edge if and only if their corresponding proteins could constitute a PPI with a confidence score larger than zero. In this case, each edge in the PPI network represented a PPI. To further indicate the strength of edges, a weight was assigned to each edge, which was the confidence score of the corresponding PPI.

### 2.3 RWR Algorithm

Based on the validated lymphoma-associated genes, we employed the RWR algorithm (Kohler et al., 2008; Macropol et al., 2009; Chen et al., 2018a; Chen et al., 2018b; Liang et al., 2020) to discover the novel genes in the PPI network. Such algorithm simulated a walker starting from one node or a set of nodes (these nodes are called seed nodes) in one network, and such walker randomly moved in the network to deliver probabilities on the seed nodes to other nodes. Given a network and *m* seed nodes, the RWR algorithm initialized a probability vector *P*_0_, with the same length as the node number of the network. One node corresponded to one component. The component of one seed node was defined as 1/*m*, and other components were set to 0. The RWR algorithm repeatedly updated such vector as follows:

$$P_{t+1}=\left(1-r\right)A^{T}P_{t}+rP_{0},$$ (1)

where *A* denotes the column-wise normalized adjacency matrix; and *r* stands for the restarting probability, which was set to 0.8 as used in some previous studies (Yuan and Lu, 2017; Zhang et al., 2017a; Zhang et al., 2017c; Chen et al., 2018a). When the vectors $P_{t+1}$ and $P_{t}$ were close enough, i.e., ${\left\|P_{t+1}-P_{t}\right\|}_{L_{1}}<10^{-6}$ , the update procedure was stopped. $P_{t+1}$ was selected as the outcome of the RWR algorithm. Based on such vector, the probability of each node, which was obtained from the seed nodes, was determined. Evidently, a node assigned with a high probability may have strong associations with the seed nodes.

In this study, the RWR program developed by Li and Patra (Li and Patra, 2010) was adopted. Although this program is designed for heterogeneous networks, we set the jumping probability to zero and selected seed nodes in one part of the network so that probabilities was transmitted only in one part of the network. Here, the 1,375 Ensembl IDs were set as the seed nodes. According to the outcome of the RWR algorithm, the nodes with high probabilities were picked up. These nodes could be the novel candidate genes related to lymphoma.

### 2.4 Screening Tests

Some candidate genes mined by the RWR algorithm were highly related to the structure of the PPI network, and these genes could induce some extreme cases. For example, some nodes may easily receive high probabilities regardless of which nodes were seed nodes. On the other hand, the candidate genes with strong associations with validated ones had higher likelihood to be novel genes related to lymphoma. In view of this, we designed three screening tests to further filter the essential candidate genes.

Permutation test. As previously mentioned, the structure of the network may influence the outcome of the RWR algorithm. To control such influence, the permutation test was adopted. We first randomly constructed 1,000 node sets, with sizes the same as that of the seed node set. The nodes in each set were fed into the RWR algorithm as the seed nodes. Then, each candidate gene selected by the RWR algorithm was also assigned a probability. After all node sets had been tested by the RWR algorithm, all candidate genes received 1,000 probabilities, and their means and standard deviations were computed. Accordingly, the Z-score was computed for each candidate gene *g* as follows:

$$Z-score\left(g\right)=\frac{Pro\left(g\right)-\mathrm{Pr}oM\left(g\right)}{\mathrm{Pr}oSTD\left(g\right)}$$ (2)

where Pro(g) denotes the probability of the candidate gene *g* obtained by using the actual seed node set; and ProM(g) and ProSTD(g) represent the mean and standard deviation of the probabilities, respectively, which were obtained by 1,000 randomly produced node sets. In statistics, the value of 1.96 is a widely accepted threshold of the Z-score to denote statistical significance. Thus, we could select candidate genes with Z-score>1.96. These genes were assigned much higher probability based on the actual seed node set than those based on randomly produced node sets, indicating their significant association with lymphoma.

Linkage test. The permutation test could decrease the influence of the PPI network. However, some candidate genes with weak or even without association with the validated genes may be included. Thus, we employed the linkage test. Several studies have reported that interacting proteins are more likely to have similar functions (Ng et al., 2010; Hu et al., 2011a; Hu et al., 2011b; Chen et al., 2016; Cai et al., 2017; Li et al., 2018; Zhang and Chen, 2020; Zhu et al., 2021). Considering the strength of the PPI, proteins that could comprise a PPI with a higher confidence score were more likely to exhibit similar functions. Hence, we adopted the interaction information mentioned in Section 2.2 to design the linkage test. For two proteins *p*_1_ and *p*_2_, their confidence score was defined as $Q\left(p_{1},p_{2}\right)$ . The maximum linkage score (MLS) was computed for each candidate gene *g* as follows:

MLS(g)=Max{Q(g,g′):g′ is a validated lymphoma associated gene} (3)

Candidate genes with high MLSs evidently had high probabilities to be novel lymphoma-associated genes and thus should be selected. Considering that 900 is the threshold of the highest confidence in STRING, we adopted such value to screen the essential candidate genes, i.e., candidate genes with MLSs no less than 900 were selected.

Enrichment test. Finally, we used the enrichment test to evaluate the importance of the candidate genes with functional terms, including gene ontology (GO) terms and KEGG pathways. The validated lymphoma-associated genes should have some similar functional terms. If a candidate gene had functional terms that were also shared by one validated lymphoma-associated gene, such gene had a high probability to be a novel lymphoma-associated gene. The enrichment score (Carmona-Saez et al., 2007) was adopted to evaluate the linkage between one gene and one GO term or KEGG pathway. The enrichment score between a gene *g* and one GO term/KEGG pathway *F* was computed as follows:

$$ES\left(g,F\right)=-\log_{10}\left(\sum_{k=m}^{n}\frac{\left(\begin{array}{l} M\\ k \end{array}\right)\left(\begin{array}{l} N-M\\ n-k \end{array}\right)}{\left(\begin{array}{l} N\\ n \end{array}\right)}\right),$$ (4)

where *N* and *M* denote the number of human genes and genes annotated by *F*, respectively; *n* represents the number of interacting genes of *g* reported in STRING; and *m* represents the number of common genes that can be interacted with *g* and was annotated by *F*. For a gene *g*, enrichment scores to all GO terms and KEGG pathways were put into a vector *V*(*g*). The associations of two genes *g* and g′ could be evaluated according to their vectors as follows:

$$\Phi \left(g,g\prime \right)=\frac{V\left(g\right)\cdot V\left(g\prime \right)}{\left\|V\left(g\right)\right\|\cdot \left\|V\left(g\prime \right)\right\|},$$ (5)

Similar to MLS, we could further calculate the maximum enrichment score (MES) for each candidate gene *g*, which could be computed as follows:

MES(g)=Max{Φ(g,g′):g′ is a validated lymphoma associated gene} (6)

A candidate gene assigning a high MES had a high probability to be a novel lymphoma-associated gene. We set the threshold 0.98 to select important candidate genes.

### 2.5 Functional Enrichment Analyses on Candidate Genes

To reveal the biological meaning behind the candidate genes identified by the random walk-based method, the functional enrichment analyses were performed, which was implemented by the R package *topGO* (https://bioconductor.org/packages/topGO/, v2.42.0) (Alexa and Rahnenführer, 2009). To conduct such analyses, identified genes were regarded as gene of interest and all available human genes were termed as background. The *p*-value threshold was set as 0.001 to identify significant enrichment results.

## 3 Results

**FIGURE 1:**
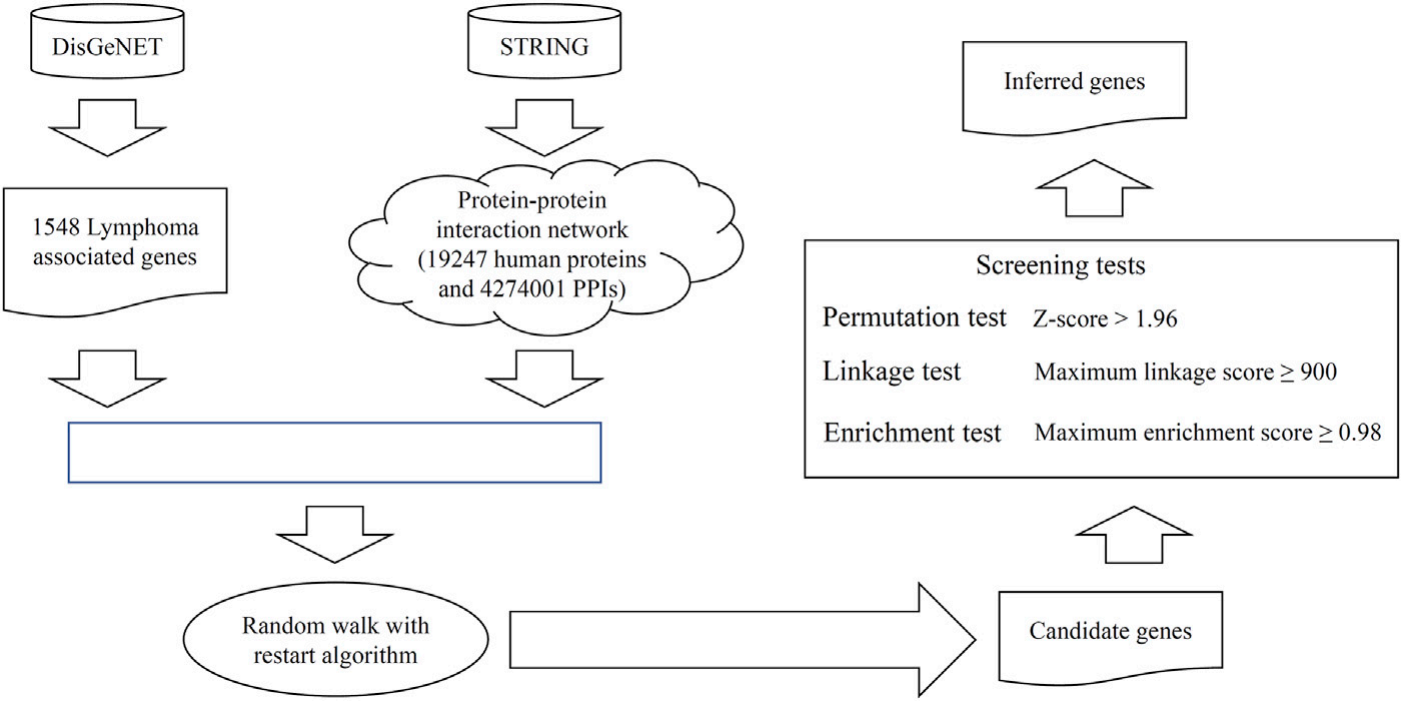
Entire procedure to mine the novel candidate genes related to lymphoma. The validated lymphoma-associated genes were retrieved from DisGeNET. From STRING, a protein–protein interaction network was constructed. These genes and the network were fed into the random walk with restart algorithm to extract the candidate genes with high probabilities. These genes were further filtered by using three screening tests to select the final inferred genes. The enrichment analysis is conducted on all inferred genes and some genes are analyzed individually.

We propose a random walk-based method to discover novel lymphoma-associated genes. The whole process is illustrated in Figure 1.

### 3.1 Results of the Random Walk-Based Method

The RWR algorithm was first performed on the PPI network with the proteins of lymphoma-associated genes as seed nodes. A probability was assigned to each node in the network to indicate its associations with the seed nodes. Nodes with probabilities no less than 10^–5^ were picked up, and their corresponding proteins were extracted. Thus, 4,962 proteins were obtained and are listed in Supplementary Table S2. The permutation test assigned a Z-score to each protein, and the scores are also listed in Supplementary Table S2. Proteins with Z-scores>1.96 were selected, resulting in 1,144 proteins. Afterward, these proteins were fed into the linkage test. Each protein was assigned an MLS, which is also provided in Supplementary Table S2. A total of 986 proteins were with MLSs no less than 900 and were selected. Finally, the enrichment test was performed to evaluate the importance of the remaining proteins. An MES was computed for each protein, and the results are listed in Supplementary Table S2. After setting the threshold of MES to 0.98, 108 proteins were obtained, which are the first 108 proteins in Supplementary Table S2. Their corresponding genes were selected and deemed to have strong associations with lymphoma. These genes are provided in Supplementary Table S3. In the following text, these genes were termed as inferred genes.

### 3.2 Associations Between Inferred Genes and Validated Genes

**FIGURE 2:**
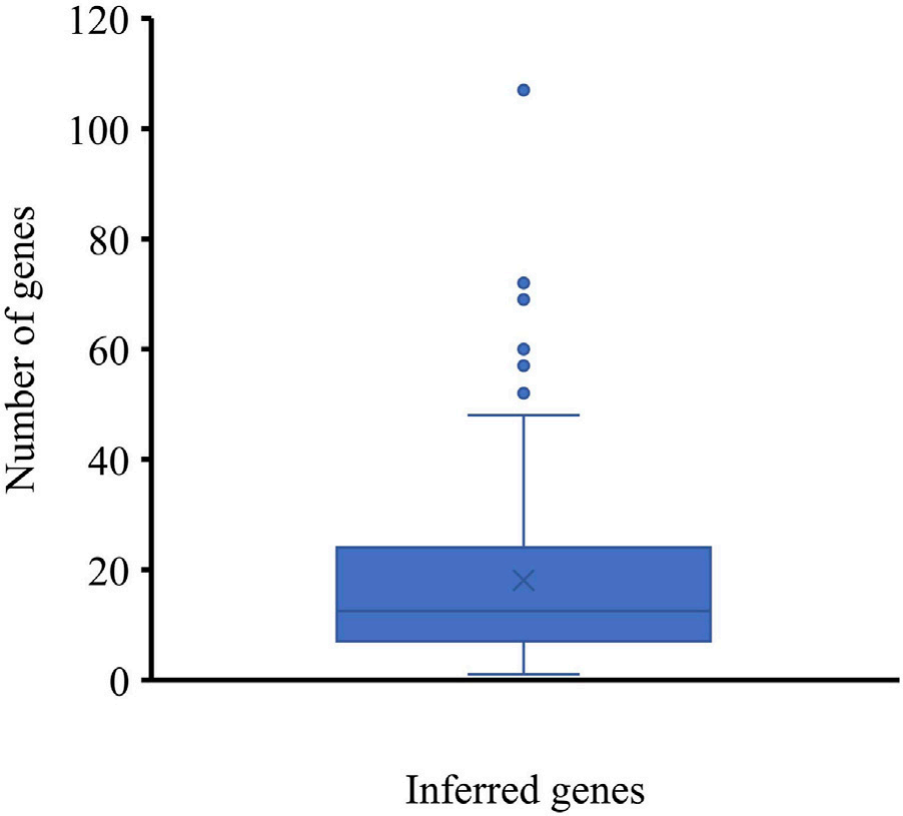
Box plot of the number of interacting lymphoma-associated genes with high confidence scores of inferred genes. Several genes can interact with over twenty lymphoma-associated genes with high confidence scores (≥900), indicating the strong associations between inferred genes with lymphoma-associated genes.

To indicate the reliability of the inferred genes, we conducted the following investigations. For each inferred gene, the number of its interacting lymphoma-associated genes with confidence scores no less than 900 was counted and is shown in a box plot (Figure 2). Some inferred genes have numerous interacting lymphoma-associated genes with confidence scores no less than 900, indicating their high relation to lymphoma. The average number of interacting lymphoma-associated genes with high confidence scores was 18.88 inferred genes, occupying 81.48%, can interact with more than five lymphoma-associated genes with high confidence score (≥900). These results implied that some hidden lymphoma-associated genes may be included in the inferred genes.

### 3.3 Enrichment Analysis on Inferred Genes

**FIGURE 3:**
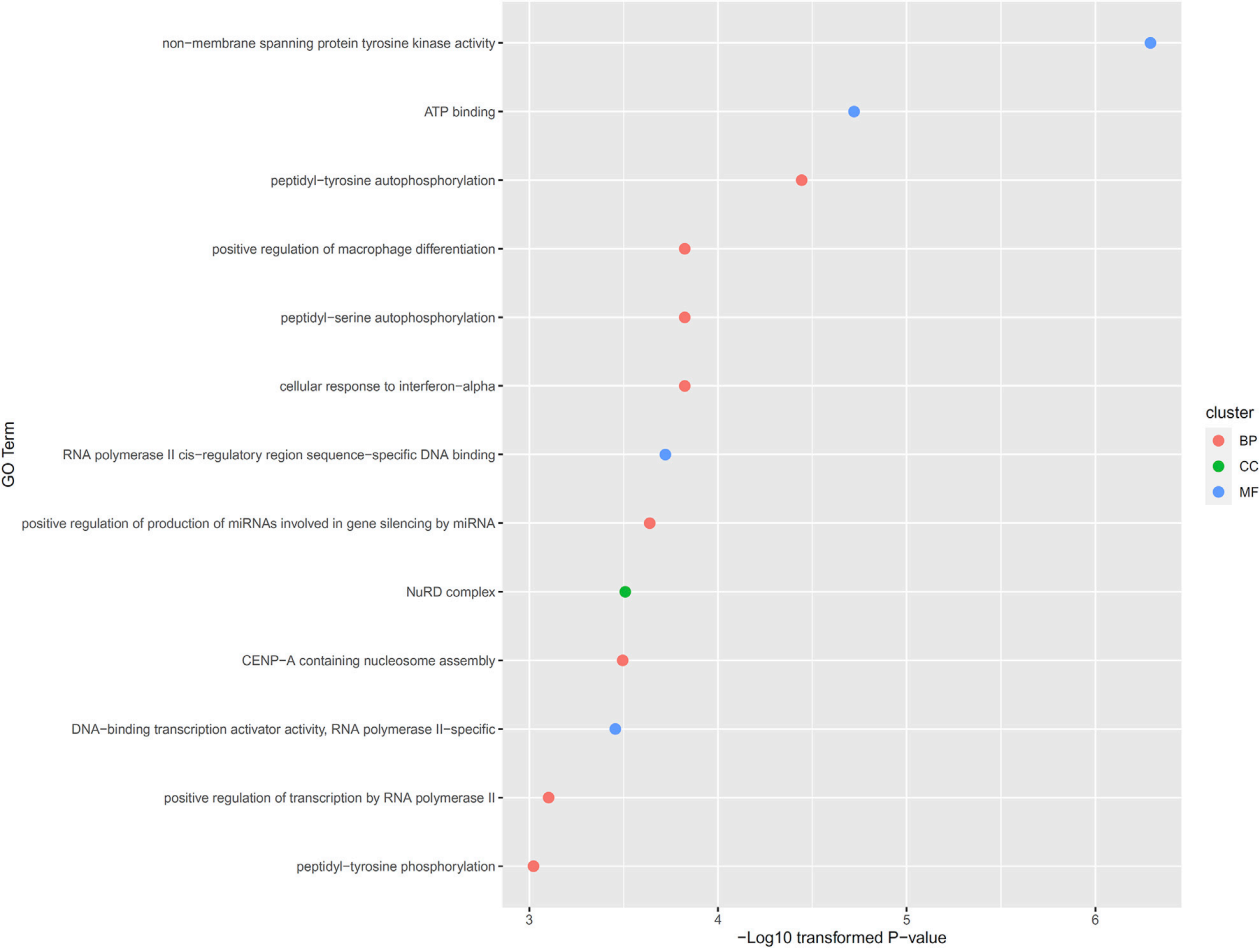
Enriched gene ontology (GO) terms on inferred genes. Thirteen GO termed are enriched on 108 inferred genes, including eight biological processes (BP) terms, four molecular function (MF) terms and one cellular component (CC) term.

Of the 108 inferred genes, we conducted functional enrichment analysis on them. Thirteen GO terms were identified with significant *p*-value less than 0.001, including eight biological processes (BP) terms, four molecular function (MF) terms and one cellular component (CC) term. Detailed information of these thirteen GO terms and their *p*-values were illustrated in Figure 3. In Section 4.2. some discussions were performed.

## 4 Discussion

From the random walk-based method, we identified a group of inferred genes that may be functionally associated with the initiation and progression of lymphoma. This section conducted some discussions to confirm their associations with lymphoma.

### 4.1 Individual Analysis on Some Inferred Genes

According to some publications, we found reliable literatures that supported the contribution of some inferred genes on lymphoma, and these genes are listed in Table 1.

**TABLE 1:** Some important inferred lymphoma-associated genes.

Ensembl id	Gene symbol	Description	Probability	Z-score	Maximum linkage score	Maximum enrichment score	Reference
ENSP00000304283	RAC3	Rac Family Small GTPase 3	9.700E−05	5.0457	998	0.9984	Coste et al. (2006)
ENSP00000370912	TEC	Tec Protein Tyrosine kinase	2.900E−05	3.2291	990	0.9979	Watson et al. (1970; Zilberman et al. (2004)
ENSP00000256458	IRAK2	Interleukin 1 Receptor Associated kinase 2	3.530E−05	3.3867	999	0.9976	Wang et al. (2014), Seltzer et al. (2020)
ENSP00000390651	IRAK4	Interleukin 1 Receptor Associated kinase 4	4.190E−05	4.3253	999	0.9967	Wang et al. (2014), Seltzer et al. (2020)
ENSP00000306124	PRKCE	Protein kinase C Epsilon	4.260E−05	3.9254	984	0.9967	Rahmatpanah et al. (2006), Wang et al. (2012)
ENSP00000261233	IRAK3	Interleukin 1 Receptor Associated kinase 3	3.540E−05	3.4101	999	0.9965	Wang et al. (2014), Seltzer et al. (2020)
ENSP00000332973	SMAD3	SMAD Family Member 3	8.280E−05	7.1221	999	0.9965	Park et al. (2001), Nakahata et al. (2010)
ENSP00000259089	BLK	BLK Proto-Oncogene, Src Family Tyrosine kinase	4.630E−05	6.2082	983	0.9964	Petersen et al. (2014)
ENSP00000264316	TXK	TXK Tyrosine kinase	2.890E−05	2.6307	915	0.9963	Liu et al. (2020)
ENSP00000263125	PRKCQ	Protein kinase C Theta	4.750E−05	4.8540	999	0.9963	Rosenwald et al. (2003)

The first gene is *RAC3* (ENSP00000304283), which had been associated with B-cell lymphoma. Early in 2006, researchers from France confirmed that the absence of *RAC3* can trigger the initiation and progression of B-cell lymphoma (Coste et al., 2006), reflecting the potential association between *RAC3* and lymphoma.

The next gene is *TEC* (ENSP00000370912). In 2004, *TEC* has been shown to mediate the abnormal proliferation and apoptosis of lymphoma cells (Zilberman et al., 2004). In 2015, another member of the *TEC* family, *BTK* has been shown to be an effective biomarker for Hodgkin and B cell non-Hodgkin lymphoma (Watson et al., 1970).

*IRAK2* (ENSP00000256458), as the next predicted gene, has been reported to contain multiple significant variants associated with lymphoma through interactions with Toll-like receptors (Wang et al., 2014). In 2020, researchers from the University of North Carolina have validated that *IRAK2*-associated signaling pathway participates in the initiation and progression of lymphoma primarily triggered by the herpes virus (Seltzer et al., 2020). *IRAK4* (ENSP00000390651) is also a participant in the IRAK signaling pathway, which is essential for the pathogenesis of lymphoma. Therefore, predicting such gene (*IRAK4*) as another lymphoma biomarker is quite reasonable. Similarly*,* another component of the IRAK signaling pathway, *IRAK3* (ENSP00000261233), has also been identified, validating the reliability of our results.

*PRKCE* (ENSP00000306124) is the next predicted gene. According to recent publications, such gene is associated with lymphoma at different omic levels. In 2006, a methylation analyses on the small B-cell lymphoma showed that *PRKCE* is a specific methylation biomarker for different clinical outcomes and prognosis of small B-cell lymphoma (Rahmatpanah et al., 2006). Further studies on transcriptomics profiling also confirmed that *PRKCE* is a specific biomarker to identify follicular lymphoma, one of the major subtypes of non-Hodgkin lymphoma (Wang et al., 2012), reflecting the specific association between *PRKCE* and lymphoma.

*SMAD3* (ENSP00000332973), as the next predicted biomarker, has been associated with lymphoma by multiple independent publications. In 2001, *SMAD3* and its homolog, *SMAD4*, have been shown to mediate the expression of autoimmune antibodies during B-cell lymphoma (Park et al., 2001). In 2010, associations between T-cell lymphoma and *SMAD4* have also been revealed (Nakahata et al., 2010). Both T-linkage and B-linkage lymphoma have been associated with *SMAD4* or related pathways, implying the specific role of *SMAD4* during the initiation and progression of lymphoma. Other inferred genes, such as *BLK* (ENSP00000259089) (Petersen et al., 2014), *TXK* (ENSP00000264316) (Liu et al., 2020), and *PRKCQ* (ENSP00000263125) (Rosenwald et al., 2003), have also been associated with lymphoma.

Thus, some inferred genes can be validated to be associated with lymphoma-related biological processes, confirming that the inferred genes discovered in this study were quite reliable.

### 4.2 Analysis of Enrichment Results on Inferred Genes

As described in Section 3.3, thirteen GO terms were identified, which were enriched by 108 inferred genes. Generally, these GO terms should be associated with the pathogenesis of lymphoma. The enriched GO terms can be further divided into two groups: transcription regulation associated GO terms and immune associated GO terms. There are multiple enriched terms associated with RNA polymerase II (RNA polymerase II *cis*-regulatory region sequence-specific DNA binding, DNA binding transcription activator activity, RNA polymerase II-specific and positive regulation of transcription by RNA polymerase II). RNA polymerase II has been widely reported to be associated with the pathogenesis of lymphoma (Kawahata et al., 1983; Devaiah et al., 2012). As for another group of GO terms, there are multiple immune responses associated GO terms, including positive regulation of macrophage differentiation and cellular response to interferon-alpha. According to recent publications, macrophage differentiation (Kant et al., 2013; Arlt et al., 2020) and interferon-alpha (Hermine et al., 2002) associated immune responses have both been reported to be associated with the pathogenesis of lymphoma.

## 5 Conclusion

In this study, a random walk-based computational method was proposed to determine the novel lymphoma-associated genes. Based on the powerful RWR algorithm and three screening tests, 108 inferred genes were obtained. The analytical results showed that some of these genes (*RAC3*, *TEC*, *IRAK2/3/4*, *PRKCE*, *SMAD3*, *BLK*, *TXK*, *PRKCQ*) could be novel lymphoma-associated genes. These findings may give new insights to investigate lymphoma and improve the understanding on the pathogeny of lymphoma.

## Data Availability

Publicly available datasets were analyzed in this study. This data can be found here: https://www.disgenet.org/.
